# Swimming faster despite obstacles: a universal mechanism behind bacterial speed enhancement in complex fluids

**DOI:** 10.15698/mic2022.07.781

**Published:** 2022-07-04

**Authors:** Shashank Kamdar, Xiang Cheng

**Affiliations:** 1 Department of Chemical Engineering and Materials Science, University of Minnesota, Minneapolis, MN 55455, USA.

**Keywords:** Bacterial motility, complex fluids, fluid mechanics, Escherichia coli, colloids

## Abstract

Bacteria constitute about 15% of global biomass and their natural environments often contain polymers and colloids, which show complex flow behaviors. It is crucial to study their motion in such environments to understand their growth and spreading as well as to design synthetic microswimmers for biomedical applications. Bacterial motion in complex viscous environments, although extensively studied over the past six decades, still remains poorly understood. In our recent study combining experimental data and theoretical analysis, we found a surprising similarity between bacterial motion in dilute colloidal suspensions and polymer solutions, which challenged the established view on the role of polymer dynamics on bacterial speed enhancement. We subsequently developed a physical model that provides a universal mechanism explaining bacterial speed enhancement in complex fluids.

Bacteria are the most abundant swimmers in nature. Motility determines the fitness and ability of their species to survive and spread in environments. They live in varied habitats, ranging from marine to soil ecosystems and to the human microbiome, which usually contain polymer molecules and microscopic particles. These microscopic objects impart complex fluid properties such as shear thinning, shear thickening and viscoelasticity, which profoundly affect the locomotion of bacteria. Understanding bacterial locomotion in such complex environments is of immense importance for deciphering key biological processes from disease infection to fertility and reproduction, and to ecosystem health. Additionally, physical insights gained from bacterial swimming in complex environments provides useful design considerations for biomimicking artificial microswimmers for drug delivery and microsurgeries. This broad range of scientific interests and engineering applications makes the study of motility a fertile ground and a natural junction for biophysicists, mathematicians, and engineers.

While the hydrodynamics of bacterial swimming in simple Newtonian fluids have been well established, our understanding of their motion in complex fluids is still far from complete. Pioneering experiments more than sixty years ago observed an increase in bacterial speeds in viscous polymeric media. Initially, this counterintuitive effect was credited to the change in flagellar conformation and structure that determine the propulsion force. However, it was later shown that increased mechanical forces due to high viscosity barely change the conformation of elastic flagella. Subsequent works have attributed the effect to a) formation of bacteria-sized pores that results in differential viscosity for the rotational and translational motion of bacteria, b) presence of small metabolizable impurities that increase bacterial activity, c) shear thinning of polymeric fluid next to fast-rotating flagella, and d) elastic deformation of polymeric chains due to flagellar rotation. Hence, even after six decades of research, it was still unclear what the physical origin of their motility enhancement in polymeric fluids is. Crucially, the role of discrete interactions between individual bacteria with dispersed polymers (or particles) has not been explored.

We revisited the long-standing puzzle with a new perspective by exploring bacterial locomotion in colloidal suspensions, instead of polymer solutions. Due to the well-controlled size and the hard-sphere nature of colloids—two valuable properties of colloidal suspensions lacking in polymer solutions—we could examine the size range of colloids from 10 nm to 500 nm and visualize discrete interactions between individual bacteria and single colloids. Surprisingly, we found that flagellated bacteria show similar motile behaviors in dilute colloidal suspensions and polymer solutions. Specifically, we uncovered a universal particle-size-dependent motility enhancement up to 80% accompanied by a strong decay of bacterial wobbling. Collectively, our experiments showed that all the quantifiable swimming features of bacteria in dilute colloidal suspensions agree quantitatively with those in dilute polymer solutions. These features include: (i) the non-monotonic behavior of bacterial swimming speed with an increase in volume fraction of colloids (or concentration of polymer); (ii) the correlation between bacterial swimming speed and the size of colloids (or equivalent hydrodynamic radius of polymer coils); (iii) the anticorrelation between bacterial swimming speed and wobble angle; (iv) the decrease of the rotational diffusivity of bacteria with an increase in volume fraction of colloids (or concentration of polymer); (v) the nearly independence of the rotational diffusivity of bacteria on the size of colloids (or equivalently the size of polymer coils); (vi) the increase of the run time of bacteria with an increase in volume fraction of colloids (or concentration of polymer); (vii) the increase of the tumble time of bacteria with an increase in volume fraction of colloids (or concentration of polymer). The comprehensive evidence above hints towards common underlying mechanism(s) that dictate the swimming of bacteria in dilute complex fluids including both colloidal suspensions and polymer solutions. However, as colloids used in our work are not attractive, they do not form gel-like networks or show shear thinning and elastic deformations. As a result, none of the previously proposed mechanisms based on unique polymer dynamics is valid in explaining our new finding.

Our experiments suggested the importance of colloidal nature of complex fluids and the discrete interactions between bacteria and microscopic objects, which result in simultaneous increase in speed and decrease in wobble angle of bacteria. Based on our experimental data, we developed a simple mathematical model to explain bacterial speed enhancement in complex fluids. When a bacterium swims close to a particle or a polymer coil, the hydrodynamic interaction between the bacterium and the particle leads to a boundary-induced torque on the bacterial body. The torque on the body is balanced by a restoring torque, which reduces the misalignment between the flagellar bundle and the cell body and therefore decreases the wobbling of the bacterium. This simple torque balance provides a parameter-free expression quantitatively predicting bacterial speed enhancement in both colloidal suspensions and polymer solutions. As a key part of the model, a new mechanism of bacterial wobbling was proposed, which successfully reproduces bacterial helical trajectories with large pitches—another mystery of bacterial locomotion. Thus, our study solved two long-standing problems at once, i.e., the origin of bacterial motility enhancement in complex fluids and the mechanism of bacterial wobbling in Newtonian fluids. Revealing the mechanism behind speed enhancement provides a reliable method that may be used to modulate swimming of artificial microswimmers in complex biological environments.

There remain questions to be answered. 1) Why do bacteria show peak speeds around similar volume fractions? 2) How do many-body interactions determine their swimming behaviors in more concentrated polymer solutions? Future experiments with polymers of well-controlled sizes would be useful to address these questions.

